# Associations between vitamin D, immunoglobulin E concentrations, and obesity

**DOI:** 10.3389/fnut.2023.1147407

**Published:** 2023-03-30

**Authors:** Angelica Avila Castillo, Tobias Hagemann, Anne Hoffmann, Ronny Baber, Ronald Biemann, Kerstin Wirkner, Sontje Krupka, Michael Stumvoll, Matthias Blüher, Nora Klöting

**Affiliations:** ^1^Medical Department III–Endocrinology, Nephrology, Rheumatology, University of Leipzig Medical Center, Leipzig, Germany; ^2^Helmholtz Institute for Metabolic, Obesity and Vascular Research (HI-MAG) of the Helmholtz Zentrum München, University of Leipzig and University Hospital Leipzig, Leipzig, Germany; ^3^LIFE-Leipzig Research Center for Civilization Diseases, University of Leipzig, Leipzig, Germany; ^4^Institute of Laboratory Medicine, Clinical Chemistry and Molecular Diagnostics (ILM), University Hospital Leipzig, Leipzig, Germany; ^5^German Center for Diabetes Research (DZD e.V.), München-Neuherberg, Germany

**Keywords:** 25(OH)D, vitamin D, IgE, adipose tissue, BMI, obesity, SAT, VAT

## Abstract

The prevalence of allergies and obesity has been increased in parallel. Low vitamin D [25(OH)D] levels have been linked to both higher body mass index (BMI) and allergies. Since the activation of the 25(OH)D receptor inhibits IgE production and 25(OH)D influences the IgE response specifically, we tested the hypothesis that circulating 25(OH)D concentrations are negatively related to circulating allergen-specific IgE concentrations distinctly in a large adult population-based study cohort. Moreover, we studied VDR gene expression in paired biopsies of abdominal subcutaneous (SAT) and visceral adipose tissue (VAT). We investigated whether magnetic resonance imaging-estimated visceral (VFM) and subcutaneous fat mass (SFM) are related to 25(OH)D levels. We found gender differences in circulating 25(OH)D and IgE concentrations. Participants with obesity showed lower 25(OH)D concentrations and higher IgE concentrations were detected in women only. Interestingly, participants with high levels of 25(OH)D are leaner and have improved glucose metabolism. In women, 25(OH)D correlate significant with VFM and SFM. *VDR* expression is significantly higher expressed in VAT and is positive associated with circulating 25(OH)D concentration. There was no association between serum IgE and 25(OH)D in the entire cohort. Based on these data, we could confirm that low levels of 25(OH)D are linked to higher BMI but could not prove our hypothesis because there is no relationship between 25(OH)D and IgE in adults. Women with higher BMI tend to have higher IgE levels what may have clinical relevance. The association between obesity and circulating 25(OH)D/IgE is not straightforward, and further knowledge is needed.

## 1. Introduction

Obesity and allergies are frequent and emerging health problems. In the last few decades, the prevalence of obesity and allergic diseases is increasing worldwide in adults and children (1–4). Several experimental and clinical studies suggested relationships between obesity, allergies and asthma, however the underlying mechanisms linking these diseases are still not well understood (5–8). Vitamin D is an important regulator of the immune system, and low vitamin D status has been associated with several immune-mediated diseases such as multiple sclerosis, inflammatory bowel disease, and allergic asthma (9–13).

There is growing evidence for an important role of vitamin D3 in the regulation of IgE and the development of allergic sensitization (14). The effects of vitamin D on lung development and the immune system may modulate the severity and course of allergic diseases (15). Vitamin D deficiency is prevalent worldwide and may partly explain the increase in asthma and allergic diseases that have occurred over the last few decades (14, 16, 17). Interestingly, vitamin D3 supplementation did not improve the time to severe exacerbation in children with asthma and low vitamin D levels (10). Multiple different mechanisms have been described that regulate serum IgE levels (18, 19). Among several IgE modulating mechanisms, vitamin D may represent an essential factor that regulates serum IgE concentrations *in vivo* (20). Activation of the vitamin D receptor (VDR) inhibits IgE production, and B cells can synthesize calcitriol from its inactive precursor 25-hydroxyvitamin D3 [25(OH)D] upon antigenic stimulation (21–23).

Interestingly, the VDR is widely found in adipocytes, which definitively supports the effect of vitamin D metabolites in adipocytes (24). Adipose tissue is considered a major reservoir for vitamin D and pools about 73% of this total vitamin D3 (25). Visceral fat contains ca. 20% more vitamin D than subcutaneous adipose tissue (26, 27). Animal studies using VDR knockout mice gain insight into the role of vitamin D metabolism in body weight management and adipose tissue biology. VDR knockout mice are lean and resistant to diet-induced obesity due to increase in energy metabolism (28), suggesting that adipogenesis could be impacted.

Taking into account that low 25(OH)D plasma level are known to be associated with obesity and that the activation of the vitamin D receptor inhibits IgE production and 25(OH)D influences specific the IgE response, we tested the hypothesis that circulating 25(OH)D concentrations are negatively related to circulating allergen-specific IgE concentrations distinctly in an adult population-based study cohort (29). Using blood IgE levels as parameter of IgE mediated allergy, we further analyzed the relationship between obesity state and IgE/25(OH)D levels. Since adipose tissue is a major reservoir for both vitamin D and VDR expression (25), we studied VDR mRNA expression in paired samples of visceral (VAT) and subcutaneous (SAT) adipose tissue (AT) from 120 subjects with a wide range of body fat mass, fat distribution and obesity related diseases and investigated whether fat distribution impacts 25(OH)D levels.

## 2. Materials and methods

### 2.1. LIFE-adult-cohort

The LIFE-Adult-Study (29, 30) is a population-based cohort study which has completed the baseline examination of 10,000 randomly selected participants from the city of Leipzig in 2014. Most of the participants were in the age range between 40 and 79 years (30). The first follow-up phase started in October 2017 with a questionnaire-based postal interview of all participants of the baseline round (30). In addition, participants who underwent MRI at baseline were reinvited for physical examinations at the study center. The first follow-up ended in August 2021. The responsible institutional ethics board of the Medical Faculty of the University of Leipzig approved the studies (approval no: 263/09 and 201/17). The responsible data protection officer approved the data privacy and safety concept of the study. Characteristics of the LIFE-Adult-Cohort are given in Supplementary Table 1. The samples have been prepared by the team from the Leipzig Medical Biobank following standard operating procedures described elsewhere (29). IgE levels were used to quantify IgE mediated allergy incidence. Self-assessment of allergy corresponded to IgE level. 25(OH)D and IgE levels were analyzed according to manufacturer’s protocol in the Institute Laboratory Medicine, Clinical Chemistry and Molecular Diagnostics on a COBAS 8000 analyzer using an electrochemiluminescent enzyme immunometric assay (Roche Diagnostics, Mannheim, Germany) and correlated to different parameters of obesity.

### 2.2. LIFE-adult sub-cohort

In a subgroup of the LIFE-Adult-cohort comprising 1,032 men (*n* = 533) and women (*n* = 499), VAT and SAT areas were estimated from abdominal Magnetic resonance imaging (MRI) scans (see characteristics in Supplementary Table 2).

### 2.3. Leipzig obesity biobank cohort

Paired AT samples from the Leipzig Obesity BioBank (LOBB) of abdominal VAT and SAT were obtained from consecutively recruited 120 Caucasian men (*n* = 56) and women (*n* = 64) who underwent open abdominal surgery for obesity surgery, cholecystectomy, or hernia repair. The age ranged from 22.2 to 68.5 years and BMI from 24 to 76 kg/m^2^. All subjects had a stable weight without body weight fluctuations of >2% for at least 3 months before surgery. Patients with severe conditions including generalized inflammation or end-stage malignant diseases were excluded from the study. AT samples were collected during elective laparoscopic abdominal surgery as described (31, 32), immediately frozen in liquid nitrogen and stored at −80°C until further analyses. The study was approved by the Ethics Committee of the University of Leipzig (approval no:159-12-21052012) and performed in accordance with the declaration of Helsinki. Characteristics of the cohort are given in Supplementary Table 3. Paired samples were used for *VDR* gene expression analyses.

### 2.4. Assays

Plasma IgE was measured in duplicate with a human IgE enzyme immunometric assay (Antibodies online, ABIN414967). Serum 25(OH)D3 were measured by a chemiluminescent enzyme immunometric assay. Fasting blood samples were taken after an overnight fast to determine glucose, insulin, and standard laboratory parameters. Plasma insulin was measured with a two-site chemiluminescent enzyme immunometric assay for the IMMULITE automated analyzer (Diagnostic Products, Los Angeles, CA, USA).

### 2.5. Measurement of anthropometric parameters

Body mass index was calculated as weight (kilograms) divided by the square of height (meters). Waist and hip circumferences were measured and waist-to-hip ratio (WHR) was calculated. Percentage body fat was measured by bioimpedance analysis.

### 2.6. *VDR* gene expression

Human *VDR* mRNA expression was measured by quantitative real-time RT-PCR in a fluorescent temperature cycler using the TaqMan assay, and fluorescence was detected on a Quant Studio 6 detector (Applied Biosystems, Darmstadt, Germany) as described elsewhere (33). Briefly, total RNA was isolated from paired SAT and VAT samples using RNeasy Lipid Tissue Mini Kit (Qiagen, Hilden, Germany) and 2 μg RNA was reverse transcribed. From each RT-PCR, 2 μl was amplified using the Fast Advanced Mastermix Kit from Life Technologies according to the manufacturer’s instructions. The following probes were used: *VDR* (Hs00172113_m1); *hypoxanthine phosphoribosyltransferase 1* (HPRT1; Hs01003267_m1), 18sRNA (Hs99999901_s1). HPRT1 as well as 18sRNA were validated to be the most stable expressed reference genes in AT and therefore used as endogenous control genes. Expression of *VDR, 18sRNA* and *HPRT1* mRNA were quantified by using the second derivative maximum method of the TaqMan Software (Applied Biosystems) determining the crossing points of individual samples by an algorithm that identifies the first turning point of the fluorescence curve. The specificity of the amplified PCR product was further verified by agarose gel electrophoresis.

### 2.7. Statistical analyses

Data are reported as means ± SD computed with the R package arsenal (v3.6.3) (34) unless otherwise noted. Methods of statistical analyses were chosen on the basis of the design of each experiment and are indicated in the figure legends. Adjusted *p*-value < 0.05 was considered statistically significant. Statistical analyses were performed in R (v4.2.1). The significance and magnitude of the correlation analyses were calculated with a non-parametric statistical approach to identify monotonic relationships using the R package RVAideMemoire (v0.9.81.2) (35) with the Spearman correlation coefficient and a confidence interval of 0.95. Absolute rho_*Spearman*_ values > 0.2 were considered as relevant correlation. The non-parametric Kruskal–Wallis test and a *post hoc* Dunn’s test was applied to determine significance between metabolic and anthropometric parameters and/or expression values of different groups and cohorts using the R package rstatix (v0.7) (36). *P*-values were corrected for multiple testing using the Bonferroni-Holm method. The predictive potential of clinical parameters on 25(OH)D and IgE was assessed *via* stepwise generalized linear additive models from the Gaussian family and a log link with the R function glm(). For comparison of 25(OH)D status, participants of the LIFE-Adult cohort were divided into subgroups of low (≥ = 20 ng/ml) and high (>20 ng/ml) serum 25(OH)D (mean = 23.61, min = 3.00, max = 131.60) according to current guidelines suggesting a required blood 25(OH)D level of 20 ng/mL (or 50 nmol/L) to avoid health risks (37–39).

## 3. Results

### 3.1. Lower 25(OH)D serum concentrations in people with obesity

**FIGURE 1:**
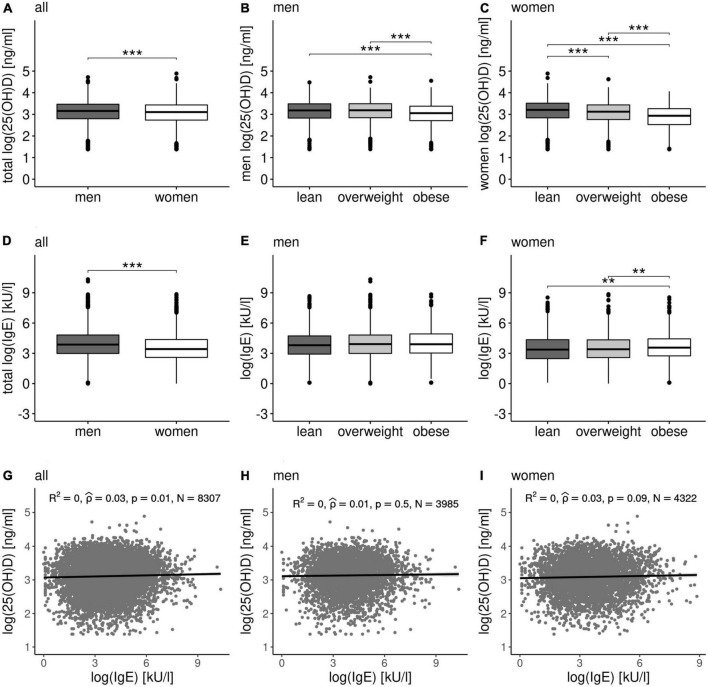
IgE and 25(OH)D concentrations and correlation studies. 25(OH)D concentrations of the entire study population of the LIFE-Adult cohort grouped by **(A)** gender (*p* < 0.001), BMI categories of **(B)** men (*p* < 0.001), and **(C)** woman (*p* < 0.001). IgE concentrations grouped by **(D)** gender (*p* < 0.001), BMI categories of **(E)** men (*p* = 0.16), and **(F)** woman (*p* = 0.003). Statistical significance was evaluated by Kruskal–Wallis and Dunn’s *post hoc* test with Bonferroni–Holm correction for multiple testing (indicated as ** <0.01 and *** <0.001). The 25(OH)D and IgE concentrations are significantly gender-specific. Significant differences were also observed in lean and overweight subjects compared to obese subjects when considering 25(OH)D concentrations in men and IgE concentrations in women. However, all BMI categories show significant differences in 25(OH)D concentration in women. No correlation is revealed between 25(OH)D and IgE concentrations in **(G)** the entire cohort (rho_*Spearman*_ = 0.03, *R*^2^ = 0, *p*-value 0.01), in **(H)** men (rho_*Spearman*_ = 0.01, *R*^2^ = 0, *p*-value 0.5), and in **(I)** women (rho_*Spearman*_ = 0.03, *R*^2^ = 0, *p*-value 0.09), respectively.

The clinical data of the LIFE-Adult cohort (*N* = 9,905) is summarized in Supplementary Table 1. Circulating IgE and 25(OH)D concentrations were significantly different between men and women as well as between people with a BMI < 25 kg/m^2^ or obesity (Figure 1). In general, men had higher circulating levels of both, IgE and 25(OH)D (Figures 1A, D). Interestingly, participants with obesity showed lower 25(OH)D levels (Figures 1B, C). In women with obesity, we detected significantly higher IgE levels (Figure 1F). However, we did not detected a significant relationship between 25(OH)D and IgE concentrations in the study population (Figures 1G–I). Additionally as shown in Table 1, the detected associations with IgE and serum 25(OH)D are very weak (|rho_*Spearman*_| < 0.2) overall with the only absolute correlation > 0.2 being the negative association of serum 25(OH)D with BMI in women. Despite low effect size, serum 25(OH)D and IgE seem to show contrary tendencies in relationship with BMI, WHR, fasting plasma insulin which is also reflected in the correlation results with 25(OH)D/IgE ratio (Table 1). Using a self-assessment of allergy, we revealed a general allergy incidence of 40.8% (4,044 out of 9,905) and food allergy incidence of 13.5% (1,344 out of 9,905).

**TABLE 1:** Correlation analysis of 25(OH)D serum concentrations and IgE with anthropometric and metabolic parameters in the LIFE-Adult cohort.

	25(OH)D all				IgE all				25(OH)D IgE ratio			
	**Both gender (*N* = 9,553)**	**Men** **(*N* = 4,577)**	**Women** **(*N* = 4,976)**	**Sign.**	**Both gender** **(*N* = 8,312)**	**Men** **(*N* = 3,988)**	**Women (*N* = 4,324)**	**Sign.**	**Both gender (*N* = 8,305)**	**Men** **(*N* = 3,984)**	**Women (*N* = 4,321)**	**Sign.**
Age (years)	0.098^###^	0.164^###^	0.032^#^	***	-0.02	-0.01	-0.031	***	0.05^###^	0.065^###^	0.044^##^	***
BMI (kg/m^2^)	-0.155^###^	-0.09^###^	-0.215^###^	***	0.055^###^	0.033	0.051^##^	***	-0.104^###^	-0.057^###^	-0.123^###^	***
WHR (ratio)	-0.028^#^	-0.055^###^	-0.105^###^	***	0.136^###^	0.067^###^	0.032	***	-0.139^###^	-0.08^###^	-0.073^###^	***
HbA1c (%)	0.01	0.035^#^	-0.02	***	0.047^###^	0.047^##^	0.029	***	-0.038^###^	-0.032	-0.029	***
FPI (ng/ml)	-0.123^###^	-0.111^###^	-0.144^###^	***	0.058^###^	0.04^#^	0.051^##^	***	-0.1^###^	-0.078^###^	-0.101^###^	***
Total cholesterol (mmol/l)	0.02	0.02	0.02	***	-0.01	0.03	0	***	0.01	-0.02	0.01	***
IgE (kU/l)	0.027^#^	0.01	0.03	***	-	-	-	***	-	-	-	***
25(OH)D (ng/ml)	-	-	-	***	0.027^#^	0.01	0.03	***	-	-	-	***

Values shown are spearman rho with respective correlation significance (^#^ < 0.05, ^##^ < 0.01, ^###^ < 0.001). Significance for differences between gender (*sign.) was calculated with Kruskal–Wallis-Test (indicated as ***p-values <0.001). WHR, waist to hip ratio; IgE, immunoglobulin E; FPI, fasting plasma insulin; sign, significance level.

### 3.2. Relationship between BMI, 25(OH)D, and IgE

To test if there is a relationship between 25(OH)D status, IgE and obesity, participants of the LIFE-Adult cohort were divided into subgroups of high (>20 ng/ml) and low (<20 ng/ml) serum 25(OH)D. Circulating 25(OH)D ranged between 3.00 and 131.60 ng/ml (mean:23.61). Participants with high levels of 25(OH)D have a lower BMI and have lower circulating glucose and insulin (Table 2). There were no differences in IgE levels when comparing subgroups with high and low levels of 25(OH)D. We further performed a stepwise generalized linear model analysis to find out what is the best predictor for 25(OH)D or IgE levels (Supplementary Table 4). We found that gender is the best predictor for IgE while gender and BMI predict 25(OH)D levels best although with lower contribution.

**TABLE 2:** Characterization of the LIFE-adult-study cohort according to low and high 25(OH)D (vitamin D) subgroups.

	Low 25(OH)D (ng/ml)				High 25(OH)D (ng/ml)			
	**Men (*N* = 1,914)**	**Women (*N* = 2,260)**	**All (*N* = 4,174)**	**Sign.**	**Men (*N* = 2,663)**	**Women (*N* = 2,716)**	**All** **(*N* = 5,379)**	**Sign.**
Age (years)	55.99 (12.71)^###^	56.84 (11.89)	56.45 (12.28)^###^	*	59.33 (12.39)	57.29 (12.41)	58.30 (12.44)	***
BMI (kg/m^2^)	28.07 (4.64)^###^	28.30 (5.92)^###^	28.19 (5.37)^###^		27.33 (3.82)	26.24 (4.64)	26.78 (4.29)	***
Waist (cm)	102.38 (13.03)^###^	94.92 (13.96)^###^	98.34 (14.04)^###^	***	100.17 (11.34)	90.10 (12.02)	95.08 (12.72)	***
WHR (ratio)	0.99 (0.07)^##^	0.88 (0.07)^###^	0.93 (0.09)^#^	***	0.99 (0.07)	0.87 (0.07)	0.93 (0.09)	***
HbA1c (%)	5.45 (0.67)^##^	5.39 (0.62)	5.42 (0.64)	*	5.45 (0.54)	5.35 (0.52)	5.40 (0.53)	***
FPG (mmol/l)	5.97 (1.33)	5.60 (1.37)^###^	5.77 (1.36)^#^	***	5.88 (1.11)	5.42 (0.94)	5.65 (1.05)	***
FPI (log ng/ml)	4.14 (0.65)^###^	4.05 (0.60)^###^	4.09 (0.62)^###^	***	4.03 (0.59)	3.90 (0.55)	3.96 (0.58)	***
Total cholesterol (mmol/l)	5.43 (1.08)	5.69 (1.08)^#^	5.57 (1.08)^#^	***	5.47 (1.04)	5.75 (1.05)	5.61 (1.06)	***
IgE (log kU/l)	3.93 (1.42)	3.49 (1.33)	3.69 (1.39)	***	3.95 (1.37)	3.53 (1.36)	3.74 (1.38)	***

Values are shown as mean (SD). Significance (sign.) is presented according to non-parametric Kruskal Wallis test between gender (* < 0.05, *** < 0.001) and between sub-groups men, women or all, respectively indicated as ^#^ < 0.05, ^##^ < 0.01, ^###^ < 0.001 in the low subgroup representative for both subgroups. Waist was measured as circumference in cm. WHR, waist to hip ratio; IgE, immunoglobulin E; FPG, fasting plasma glucose; FPI, fasting plasma insulin; sign, significance level.

### 3.3. Abdominal fat area correlates with circulating 25(OH)D, but not with IgE

**FIGURE 2:**
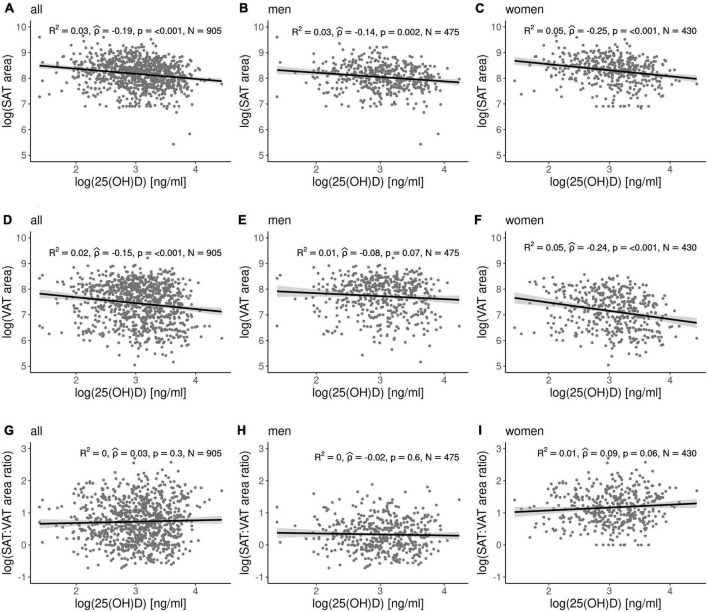
Correlation analyses between 25(OH)D and fat tissue areas. In LIFE-Adult subcohort significant correlations were observed only in women for 25(OH)D and SAT area **(A–C)** and VAT area **(D–F)**. **(G–I)** No relation was detected between SAT:VAT ratio and 25(OH)D. Fat areas were measured by means of magnetic resonance imaging.

Since we detected a relationship between 25(OH)D and obesity parameters, we further analyzed SAT and VAT fat area in more detail. In a LIFE-Adult sub-cohort of 1,032 individuals [*N* = 905 with available 25(OH)D data and *N* = 436 with available IgE data] we found significant gender differences in SAT and VAT fat area (see study characteristics in Supplementary Table 2) and related VAT and SAT areas from MRI scans to circulating 25(OH)D and IgE levels. While no clear association between 25(OH)D serum concentrations in SAT and VAT areas is found in the overall cohort (Figures 2A, D) and men (Figures 2B, E), we detected weak correlations only in women (Figures 2C, F). Also, no correlation of 25(OH)D serum concentration with SAT:VAT ratio is revealed in both gender (Figures 2G–I). Moreover, IgE levels did not correlate with any measures of fat areas (Supplementary Figure 1). We did not find correlations between 25(OH)D and IgE levels and SAT:VAT ratio (abdominal fat distribution). In both gender, negative correlations between the SAT/VAT ratio and age, BMI, waist circumference, WHR, HbA1c, total plasma glucose, and total cholesterol were found while SAT:VAT ratio also correlated negatively with plasma insulin in men (Supplementary Table 5).

### 3.4. Association between *VDR* mRNA expression in AT and parameters of obesity

Vitamin D receptor mRNA expression was studied in paired VAT and SAT samples of the LOBB sub-cohort obtained from 120 individuals compromising 56 men and 64 women with a wide range of age (22.2–68.5 years) and BMI (24–76 kg/m^2^) (see Supplementary Table 3). In both genders, *VDR* gene expression was significantly higher in VAT than in SAT (Supplementary Figures 2A–C). Whereas *VDR* expression in AT was not significantly different between men and women (Supplementary Table 3), we found a significant intercorrelation between SAT and VAT *VDR* expression (Supplementary Figure 2D; rho_*Spearman*_ = 0.64; *R*^2^ = 0.49; *p* < 0.001). *VDR* expression did not correlate with percentage of body fat (Supplementary Figures 2E, F) or 25(OH)D serum concentrations in SAT (Supplementary Figure 2G) but with 25(OH)D serum concentrations in VAT (Supplementary Figure 2H).

## 4. Discussion

Obesity is defined as a complex chronic disease, which is characterized by an excessive accumulation of body fat resulting from an imbalance between energy intake and expenditure (40–42). Low circulating 25(OH)D concentrations have been consistently associated with obesity in cross-sectional studies (43, 44). Elevated serum 25(OH)D levels may contribute to greater diet-induced weight loss response (45). There is no clear evidence for a beneficial effect of vitamin D supplementation on weight loss efficiency or changes in cardio-metabolic parameters in people with obesity (46). Data from the National Health and Nutrition Examination Survey 2005–2006 suggested that vitamin D deficiency is associated with higher levels of IgE sensitization in children and adolescents (47). However, for a long time obesity has not been considered a modulator of IgE serum concentrations (48). Only very recently, Carballo et al. (49) found in a cross-sectional study of 1,516 adults that obesity and its associated altered metabolic parameters are associated with high IgE serum concentrations. The potential interrelationship between circulating vitamin D, IgE and obesity has not been systematically studied. We tested the hypothesis that circulating 25(OH)D concentrations are negatively related to circulating allergen-specific IgE concentrations distinctly in a large adult population-based study cohort.

In 9,905 participants of the LIFE-Adult population-based study, we find that both 25(OH)D and IgE serum concentrations are gender specific. This observation is in accordance with previous reports (49–51). Whereas 25(OH)D serum concentrations weakly correlate negatively with BMI, IgE shows no correlation but a contrary tendency toward BMI (Table 1).

It has been shown that people with obesity tend to have a low vitamin D status and higher IgE levels (52). In our study, men had higher circulating levels of both, IgE and 25(OH)D. In accordance with previous reports (43, 44, 53), people with obesity were characterized by lower 25(OH)D and higher IgE concentrations. In our study, we were able to confirm these reports for 25(OH)D levels in both gender and for IgE in women (Figure 1). However, we found no direct association of circulating IgE and 25(OH)D. Also, the ratio of circulating 25(OH)D to IgE does not correlate with obesity, suggesting that 25(OH)D deficiency is not linked to obesity through increased IgE serum concentrations and therefore, we could not prove our hypothesis.

Study participants from the LIFE-Adult-cohort with higher levels of 25(OH)D are leaner (measured as BMI) and have lower circulating glucose and insulin concentrations (Table 2). Overall, we did not find strong correlations of 25(OH)D and IgE levels with clinical parameters. Merely the interaction of 25(OH)D with BMI has a reasonable effect size.

We further investigated *VDR* expression in paired samples of VAT and SAT in relation to circulating 25(OH)D and IgE. On the molecular level we find that *VDR* expression is positively related to higher 25(OH)D levels suggesting that the circulating 25(OH)D ligand regulates *VDR* mRNA expression in adipose tissue. There is evidence that vitamin D deficiency and *VDR* expression in adipocytes are linked to obesity (54). Moreover, mice with an AT specific deletion of the *VDR* mirror vitamin D-deficiency in humans and exhibit AT fibrosis and inflammation (55).

In this context, recent studies suggested that AT may be a direct target of vitamin D (52). Studies have shown major differences between SAT and VAT in the expression of vitamin D-metabolizing enzymes. The expression of the *VDR, 25-hydroxyvitamin-D-1*α*-hydroxylase (CYP27B1)* genes, and *24-hydroxylase enzymes* has been shown in most human tissues (56), and it is also highly expressed in adipocytes (57). Moreover, it was suggested that *VDR* expression is increased in people with obesity, who have more VAT than lean subjects, but the physiological relevance of this upregulation has not yet been elucidated. In our Obesity Biobank cohort study, we found that *VDR* gene expression significantly varied between fat depots. We found significantly higher *VDR* expression in human VAT than in SAT. Interestingly, we found positive correlations between VAT *VDR* expression and 25(OH)D as well as a strong intercorrelation between both fat depots SAT and VAT with *VDR*.

Taken together, we show that 25(OH)D, IgE and anthropometric parameters are interrelated in a gender specific manner.

We could confirm that low levels of 25(OH)D are linked to higher BMI but no relationship exists between circulating 25(OH)D and IgE levels in our adult cohort. Neither fat distribution nor the amount of body fat have an impact on circulating IgE concentrations while fat mass areas show negative correlation with 25(OH)D concentrations only in women. On the molecular level, elevated *VDR* mRNA expression in AT is related to higher amount of circulating 25(OH)D ligand. In a gender-dependent manner, women with higher BMI tend to have higher IgE levels what may have clinical relevance. In conclusion, these data do not clearly converge toward that circulating 25(OH)D concentrations are related to circulating allergen-specific IgE concentrations under different obesity states. More research is needed to clarify the complex interplay between 25(OH)D, IgE and obesity.

## Data availability statement

The original contributions presented in this study are included in the article/Supplementary material, further inquiries can be directed to the corresponding authors.

## Ethics statement

The studies involving human participants were reviewed and approved by the Ethics Board of the Medical Faculty of the University of Leipzig (approval nos: 263/09 and 201/17). The patients/participants provided their written informed consent to participate in this study.

## Author contributions

TH, AA, AH, NK, MB, and SK wrote and participated in all aspects of this research, including the field investigation. MS, TH, and AH performed statistical analysis. RB, RBa, and KW were involved in biobanking and laboratory measurements from the LIFE-cohort. All authors reviewed the final manuscript, interpreted the results, edited, reviewed, and supervised this research.
