# Supplementary material for: Associations between vitamin D, immunoglobulin E concentrations, and obesity
Source: Front Nutr. 2023 Mar 30;10:1147407. doi: 10.3389/fnut.2023.1147407 (PMC10097930; doi:10.3389/fnut.2023.1147407)
Supplement: Supplementary file 1 [file Data_Sheet_1.docx]

**Supplemental Figures**


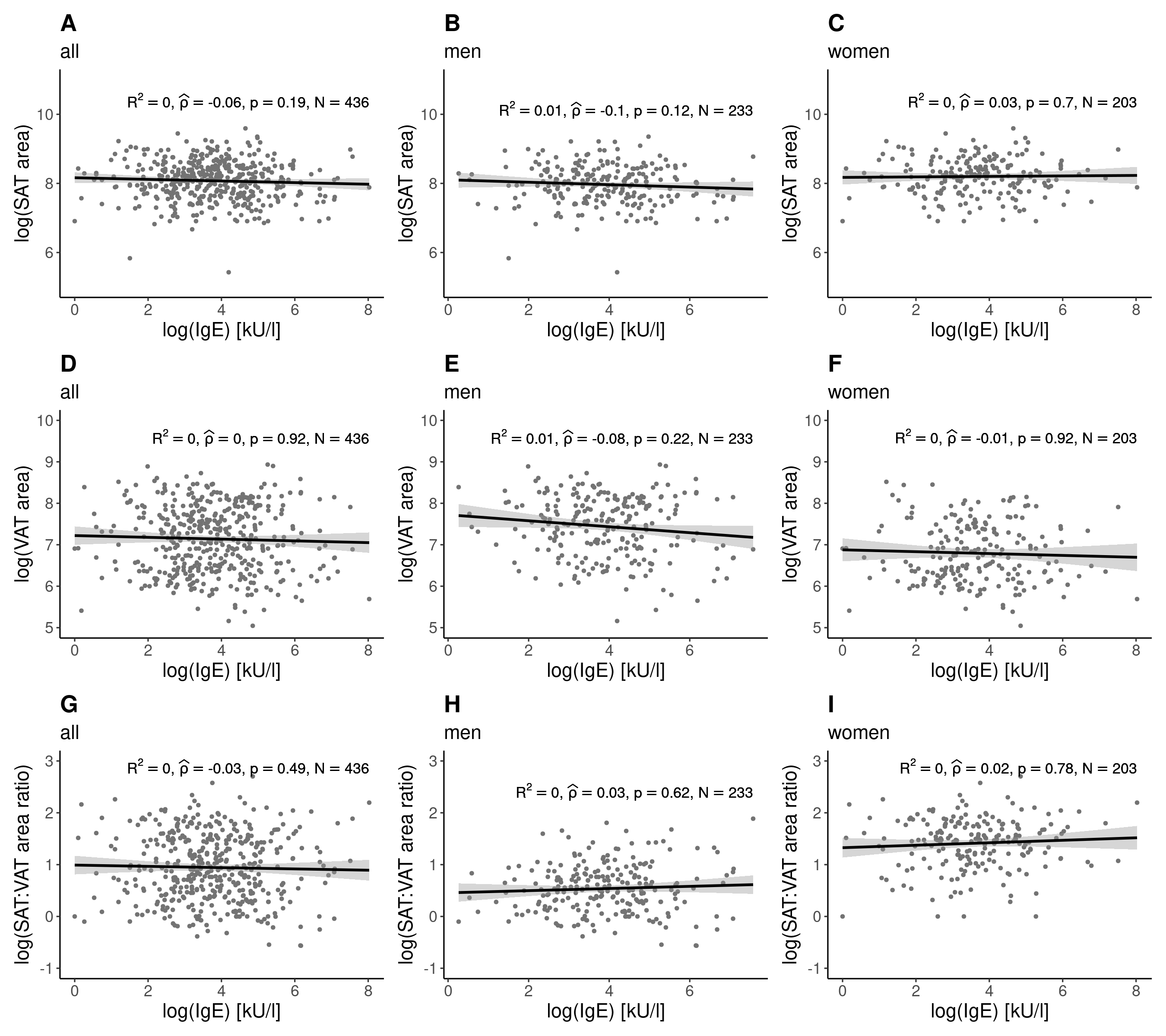


**Figure S1**. **Correlation analysis of IgE and fat tissue areas**. In LIFE-Adult subcohort IgE levels do not show any correlation with (A-C) SAT area, (D-F) VAT area or (G-I) the ratio of SAT to VAT area. Correlation coefficient shows relations with 25(OH)D in VAT (rho_Spearman_ = 0.21) but not in SAT. Fat areas were measured by means of magnetic resonance imaging.


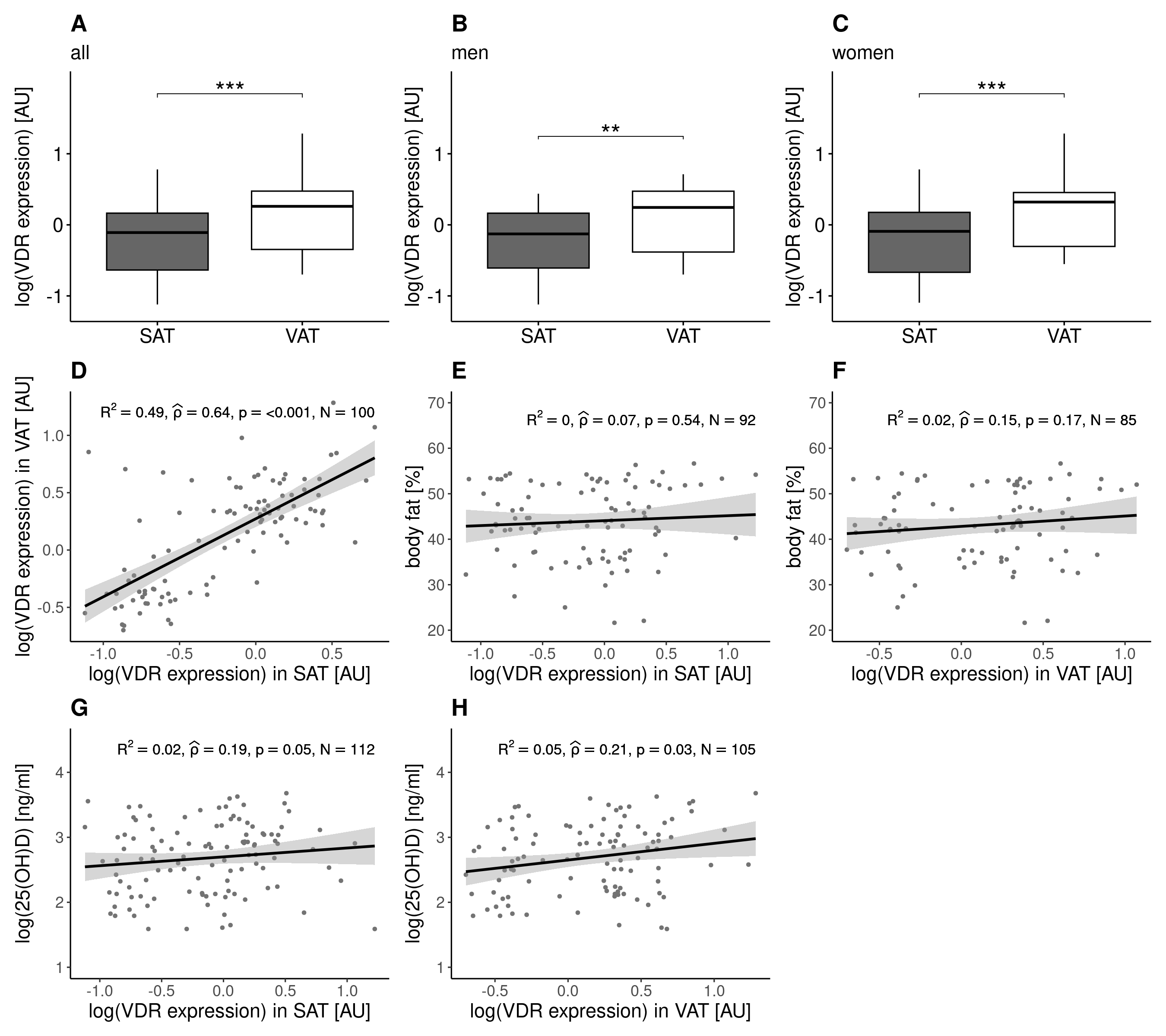


**Figure S2**. **Correlation analysis of *VDR* mRNA expression in SAT and VAT**. (A-C) VDR expression is significantly higher in VAT compared to SAT and independent of sex. Significant correlation was observed (D) in VDR expression between VAT and SAT but not for (E-F) body fat. (G-H) VDR expression correlated in VAT with 25(OH)D (rho_Spearman_ = 0.21) but not in SAT.
