# Supplementary material for: Associations between vitamin D, immunoglobulin E concentrations, and obesity
Source: Front Nutr. 2023 Mar 30;10:1147407. doi: 10.3389/fnut.2023.1147407 (PMC10097930; doi:10.3389/fnut.2023.1147407)
Supplement: Supplementary file 2 [file Data_Sheet_2.docx]

**Supplemental Material**

Table S1. Clinical characterization of the entire LIFE-Adult study cohort **(N=9905)**.

|  | **lean** | | | **overweight** | | | **obese** | | |
| --- | --- | --- | --- | --- | --- | --- | --- | --- | --- |
|  | men   (N=1,326) | women (N=2,087) | sign. | men (N=2,221) | women (N=1,798) | sign. | men  (N=1,180) | women  (N=1,293) | sign. |
| **Age (years)** | 53.98 (14.04) | 52.93 (12.63) | * | 58.89 (12.01) | 59.23 (11.52) |  | 60.69 (11.03) | 60.71 (10.55) |  |
| **BMI (kg/m²)** | 23.21 (1.40) | 22.49 (1.62) | *** | 27.27 (1.39) | 27.27 (1.45) |  | 33.31 (3.19) | 34.51 (4.29) | *** |
| **Waist circumference (cm)** | 89.08 (6.41) | 81.22 (6.81) | *** | 100.27 (6.16) | 93.20 (6.51) | *** | 116.06 (9.06) | 108.44 (9.91) | *** |
| **WHR (ratio)** | 0.93 (0.06) | 0.84 (0.06) | *** | 0.99 (0.06) | 0.89 (0.06) | *** | 1.05 (0.05) | 0.91 (0.06) | *** |
| **HbA1c (%)** | 5.27 (0.40) | 5.21 (0.48) | *** | 5.43 (0.56) | 5.36 (0.46) | ** | 5.69 (0.74) | 5.62 (0.72) |  |
| **FPG (mmol/l)** | 5.39 (1.05) | 5.58 (1.05) | *** | 5.54 (1.03) | 5.87 (1.07) | *** | 5.36 (1.09) | 5.75 (1.04) | *** |
| **FPI (log ng/ml)** | 3.67 (0.47) | 3.63 (0.46) | * | 4.05 (0.52) | 4.02 (0.48) | * | 4.59 (0.57) | 4.43 (0.52) | *** |
| **Total cholesterol (mmol/l)** | 5.49 (0.79) | 5.13 (0.90) | *** | 5.89 (1.10) | 5.52 (0.94) | *** | 6.47 (1.54) | 6.07 (1.50) | *** |
| **IgE (log kU/l)** | 3.89 (1.38) | 3.47 (1.35) | *** | 3.95 (1.39) | 3.48 (1.36) | *** | 3.99 (1.39) | 3.63 (1.33) | *** |
| **25(OH)D   (log ng/ml)** | 3.13 (0.51) | 3.16 (0.50) |  | 3.14 (0.48) | 3.09 (0.49) | *** | 3.02 (0.50) | 2.88 (0.51) | *** |

Values are shown as mean and standard deviation (SD). Significance (sign) is shown according to non-parametric Kruskal Wallis test between gender (* <0.05, ** < 0.005, *** < 0.001). Abbreviations: Waist to hip ratio, WHR; Immunoglobulin E, IgE; Fasting plasma glucose, FPG; fasting plasma insulin, FPI; significance level, sign.

**Table S2. Clinical characteristics of LIFE Adult MRI sub-cohort (N=1.032).**

|  | **men (N=533)** | **women (N=499)** | **total (N=1032)** | **sign.** |
| --- | --- | --- | --- | --- |
| **Age (years)** | 51.54 (15.73) | 51.09 (15.63) | 51.32 (15.68) |  |
| **BMI (kg/m²)** | 27.08 (4.12) | 25.96 (4.98) | 26.54 (4.59) | *** |
| **WHR (ratio)** | 0.96 (0.08) | 0.85 (0.07) | 0.91 (0.09) | *** |
| **HbA1c (%)** | 5.32 (0.53) | 5.25 (0.50) | 5.29 (0.52) | * |
| **SAT fat area (log cm²)** | 8.06 (0.49) | 8.30 (0.51) | 8.17 (0.51) | *** |
| **VAT fat area (log cm²)** | 7.75 (0.70) | 7.14 (0.72) | 7.45 (0.77) | *** |
| **IgE (log kU/l)** | 3.90 (1.33) | 3.61 (1.40) | 3.76 (1.37) | * |
| **25(OH)D (log ng/ml)** | 3.02 (0.48) | 3.03 (0.51) | 3.03 (0.50) |  |
| **FPI (ng/ml)** | 3.98 (0.64) | 3.94 (0.49) | 3.96 (0.58) |  |
| **Total cholesterol (mmol/l)** | 5.41 (1.03) | 5.60 (1.04) | 5.50 (1.04) | ** |

Values are given as mean and standard deviation (SD). Significance (sign) is shown according to non-parametric Kruskal Wallis test between gender (* <0.05, ** < 0.01, *** < 0.001). Abbreviations: Waist to hip ratio, WHR; Immunoglobulin E, IgE; fasting plasma insulin, FPI; subcutaneous adipose tissue, SAT; visceral adipose tissue, VAT; significance level, sign.

**Table S3: Clinical characteristics of the adipose tissue donors (N=120) from the Leipzig Obesity Biobank.**

|  | **men (N=56)** | **women (N=64)** | **total (N=120)** | **sign.** |
| --- | --- | --- | --- | --- |
| **Age (years)** | 46.18 (12.60) | 45.25 (10.78) | 45.69 (11.62) |  |
| **BMI (kg/m²)** | 49.87 (9.31) | 50.50 (7.34) | 50.21 (8.29) |  |
| **WHR (ratio)** | 1.07 (0.07) | 0.91 (0.07) | 0.97 (0.10) | *** |
| **HbA1c (%)** | 6.42 (1.63) | 5.51 (0.87) | 5.93 (1.35) | *** |
| **Log SAT VDR expression** | -0.17 (0.47) | -0.12 (0.55) | -0.14 (0.51) |  |
| **Log VAT VDR expression** | 0.10 (0.45) | 0.20 (0.49) | 0.15 (0.47) |  |
| **IgE (log kU/l)** | 6.77 (11.16) | 3.99 (6.69) | 5.31 (9.16) |  |
| **25(OH)D (ng/ml)** | 14.56 (7.01) | 16.84 (9.35) | 15.78 (8.39) |  |
| **FPI (ng/ml)** | 0.07 (0.26) | 0.03 (0.18) | 0.04 (0.21) |  |
| **Total cholesterol (mmol/l)** | 4.41 (1.09) | 4.27 (1.08) | 4.33 (1.08) |  |

Values are given as mean and standard deviation (SD). Significance (sign) is shown according to non-parametric Kruskal Wallis test between gender (*** < 0.001). Abbreviations: Waist to hip ratio, WHR; Immunoglobulin E, IgE; fasting plasma insulin, FPI; Vitamin D receptor, VDR; subcutaneous adipose tissue, SAT; visceral adipose tissue, VAT; significance level, sign.

**Table S4. Stepwise generalized linear modeling of 25(OH)D and IgE in the entire LIFE-Adult study cohort (N=9905).**

|  | **Circulating serum measures**  **β-coefficient in additive model** | |
| --- | --- | --- |
|  | **25(OH)D (ng/ml)** | **IgE (kU/l)** |
| **Model 1**  Age (years) | 3.539e-03 *** | 1.461e-03 |
| **Model 2**  Age (years)  Gender | 3.460e-03 ***  -0.029 ** | -1.929e-03  -0.554 *** |
| **Model 3**  Age (years)  Gender  BMI (kg/m²) | 5.338e-03 ***  -0.044 ***  -0.023 *** | 3.107e-03  -0.555 ***  0.014 |
| **Model 4**  Age (years)  Gender  Log IgE  Log 25(OH)D | 3.544e-03 ***  -0.027 *  -9.818e-03 *  - | -1.77e-03  -0.549 ***  -  3.845e-03 |

Generalized linear modeling analysis with gaussian family showing the association of circulating serum measures with age, gender and BMI for the entire LIFE-Adult-Study population (N=9905). Values shown are the computed estimates within each additive model (significance levels: * <0.05, ** < 0.005, *** < 0.001)

**Table S5: Correlation analysis of SAT:VAT ratio of LIFE Adult MRI sub-cohort (N=1.032).**

|  | SAT:VAT ratio | | | |
| --- | --- | --- | --- | --- |
|  | all  (N = 1032) | men  (N = 533) | women  (N = 499) | sign. |
| Age (years) | -0.458^###^ | -0.606^###^ | -0.638^###^ |  |
| BMI (kg/m²) | -0.302^###^ | -0.221^###^ | -0.298^###^ | *** |
| Waist Circumference (cm) | -0.491^###^ | -0.318^###^ | -0.375^###^ | *** |
| WHR (ratio) | -0.718^###^ | -0.537^###^ | -0.494^###^ | *** |
| HbA1c (%) | -0.275^###^ | -0.278^###^ | -0.367^###^ | * |
| FPI (ng/ml) | -0.173^###^ | -0.213^###^ | -0.152^#^ |  |
| FPG (mmol/l) | -0.38^###^ | -0.295^###^ | -0.352^###^ | *** |
| Total cholesterol (mmol/l) | -0.182^###^ | -0.259^###^ | -0.398^###^ | * |
| 25(OH)D (log ng/ml) | 0.034 | -0.024 | 0.089 |  |
| IgE (log kU/l) | -0.033 | 0.032 | 0.02 | * |

Values shown are spearman rho with respective correlation significance ( ### < 0.001). Significance for differences between gender (sign.) was calculated with Kruskal-Wallis-Test between gender (indicated as * <0.05, *** < 0.001). Abbreviations: WHR, waist to hip ratio; Immunoglobulin E, IgE; fasting plasma insulin, FPI; Fasting plasma glucose, FPG; significance level, sign.
